# Cross-Sectional Analysis of Overall Dietary Intake and Mediterranean Dietary Pattern in Patients with Crohn’s Disease

**DOI:** 10.3390/nu10111761

**Published:** 2018-11-14

**Authors:** Lorian Taylor, Abdulelah Almutairdi, Nusrat Shommu, Richard Fedorak, Subrata Ghosh, Raylene A. Reimer, Remo Panaccione, Maitreyi Raman

**Affiliations:** 1 Department of Medicine, University of Calgary, Calgary, AB T2N 4N1, Canada; lorian.taylor@ucalgary.ca (L.T.); abdulelah.almutairdi@ucalgary.ca (A.A.); nsshommu@ucalgary.ca (N.S.); rpanacci@ucalgary.ca (R.P.); 2 Faculty of Medicine & Dentistry, University of Alberta, Edmonton, AB T6G 2R7, Canada; richard.fedorak@ualberta.ca; 3 Institute of Translational Medicine, NIHR Biomedical Research Centre, University of Birmingham and Birmingham University Hospitals, Birmingham B15 2TT, UK; s.ghosh@bham.ac.uk; 4 Faculty of Kinesiology, University of Calgary, Calgary, AB T2N 4N1, Canada; reimer@ucalgary.ca

**Keywords:** Crohn’s disease, dietary intake, malnutrition, Mediterranean diet

## Abstract

The primary objective of this study was to explore the macro- and micro-nutrient intakes and dietary patterns of patients with Crohn’s disease (CD). Secondary objectives were to (a) compare the micronutrient intakes of CD patients with a representative sample of individuals, (b) describe the macro- and micronutrient intakes of male and female CD patients, and (c) describe Mediterranean diet scores (P-MDS) of male and female CD patients in remission that were recruited from an inflammatory bowel disease (IBD) clinic in Calgary, AB. Consecutive patients with ileal and/or colonic CD in endoscopic remission were recruited for participation in this cross-sectional study. Sixty-seven patients were enrolled with a mean age of 45, and a Body Mass Index (BMI) ≥ 25. Compared with the representative sample, patients with CD had similar energy, protein, carbohydrate, and total fat intake. However, polyunsaturated fats (PUFA), omega-6 and 3, and monounsaturated fats (MUFA) were lower in CD patients and dietary fiber intake was higher (*p* < 0.05). Vitamins C, D, thiamin, niacin, magnesium, phosphorus, zinc, and potassium were all significantly lower in all CD patients when compared to the representative sample (*p* < 0.05). Few patients with CD met the P-MDS criteria and overall scores were low (mean 4.5, Standard Deviation (SD) = 1.1 in males and 4.7, SD = 1.8 in females). The CD patients in this study had suboptimal dietary intakes and patterns and these data may be used to inform future dietary interventions in this population to improve intake.

## 1. Introduction

There is a compelling argument for environmental factors such as diet to play a role in the course of inflammatory bowel disease (IBD) [1]. Given the mounting evidence for the role of diet and gut microbial composition and function in IBD development and exacerbation, a richer understanding of the role of diet in disease pathogenesis is warranted. Perturbations related to dietary intake are thought to relate to the consumption of a dietary pattern that negatively alters gut microbiota composition [2,3], leading to functional changes in short chain fatty acid (SCFA) profiles affecting the inflammation process [4] and stimulating an inappropriate immune activation of the gut mucosa.

There are limited published data describing the dietary patterns and macro- and micronutrient intakes in patients with Crohn’s disease (CD); in particular, the differences between males and females. Nearly 50% of CD patients perceive diet to be an initiating factor in their disease, and over half of patients have reported diet exacerbates disease severity [5]. In IBD, deficiencies of micro- and macronutrients are observed and arise through multifactorial etiologies including disruptions in digestion, malabsorption, and disease activity, resulting in increased energy and nutrient requirements, anorexia, consumption of a nondiversified diet with food avoidance due to symptoms, and cachexia arising as a consequence of pro-inflammatory cytokines [6]. The reported prevalence of malnutrition is variable (12–85%) [7,8,9] and depends on disease activity and the definitions used to define malnutrition. Nutrient deficiencies usually develop over time and are linked to duration of illness. Macronutrient deficiencies leading to protein-energy malnutrition are less common in the biologic era, however, they are still of significant prevalence in patients admitted to hospital. Overt malnutrition may lead to complications such as frequent infections, poor immunity, and increased hospitalizations [10]. Micronutrient deficiencies occur for multiple reasons including chronic blood loss leading to iron deficiency; chronic diarrhea resulting in hypomagnesemia or zinc deficiency; malabsorption leading to B12, folate, and vitamin D deficiencies; and other deficiencies such as antioxidants [11,12]. No previous study has reported the prevalence of macro- and micronutrient deficiencies in Canadian CD patients compared to a representative population.

While single nutrients may play a role in the natural history of IBD [13,14], single nutrients are rarely consumed in isolation. Specific to IBD, epidemiological studies have described associations of increased risk of IBD and high dietary intake of refined sugar, grains, calories, animal fat from meat and dairy, and regular intake of processed foods, while a diet high in fruit, vegetables, and dietary fiber decreases risk [15,16]. A recent meta-analysis showed an inverse association between the intake of vegetables and fruit and CD, respectively (pooled odds ratio for the highest versus lowest consumption of fruit = 0.57; 95% Confidence Interval (CI) 0.44–0.74) [17]. Patients consuming a pro-inflammatory diet (e.g., high in animal protein, low in fruit and vegetables) have demonstrated a higher risk of ulcerative colitis [18]. Recently, the association between food intake and course of disease in patients with IBD was explored using fecal calprotectin and a protective role of legumes and potato, with a detrimental influence of meat in maintaining clinical remission in IBD was identified [19].

There is a large body of evidence showing Mediterranean dietary patterns regulate inflammation in chronic disease [20,21,22]. A Mediterranean dietary pattern is high in extra-virgin olive oil, vegetables, fruit, legumes, nuts, and seeds with a moderate consumption of fish, poultry, and milk products and is low in processed foods, baked goods, and red and processed meat. This dietary pattern is high in monounsaturated fats (MUFA), omega-3 polyunsaturated fats (PUFA), fermentable fibers and polyphenols, and adherence to a Mediterranean dietary pattern is associated with lower levels of inflammation biomarkers. A Mediterranean dietary pattern has shown positive effects in chronic disease [23], although it may impact men and women differently [24], and has been understudied in IBD. 

The primary objective of this study was to examine the macro- and micro-nutrient intakes and dietary patterns of patients with CD to inform future dietary interventions. Secondary objectives were to (a) compare micronutrient intakes of CD patients to a representative sample of individuals, (b) describe macro- and micronutrient intakes of male and female CD patients, and (c) describe the Mediterranean diet scores (P-MDS) of male and female CD patients in a Canadian ambulatory care gastroenterology clinical setting.

## 2. Methods

### 2.1. Study Design and Recruitment

A single center cross-sectional study was undertaken at the University of Calgary, Foothills Medical Center, in Calgary, AB, Canada. Eligible patients were recruited consecutively from ambulatory gastrointestinal clinics at this site. 

### 2.2. Eligibility Criteria

To be eligible for the study, patients were required to: (a) be ≥18 years of age; (b) have a documented diagnosis of ileal and/or colonic CD based on clinical, radiological and endoscopic criteria; (c) be in clinical and endoscopic remission, defined by a Harvey Bradshaw Index (HBI) <5 with evidence of mucosal healing by endoscopy defined by no large ulcers, inflammation, or strictures, within three months of recruitment; (d) been in steroid-free remission for three months prior to study entry; (e) achieved induction of remission either through anti-tumor necrosis factor (anti-TNF) agents or corticosteroids; and (f) provide informed consent. Patients were excluded if: (a) HBI >5, (b) had evidence of active endoscopic mucosal disease, (c) used corticosteroids within the preceding three months, (d) had >1 bowel resection, (d) presence of ostomy, (e) used laxatives in the past 3 months, (f) used prebiotic fiber supplements in the past three months, (f) used probiotic supplements in the past three months, and (g) used antibiotics in the past six months.

### 2.3. Ethics and Consent

The study protocol was approved by the University of Calgary Conjoint Health Research Ethics Board REB15-1805. All participants provided informed written consent prior to participating in the study.

### 2.4. Data Collection

Patient characteristics, medications, and symptoms were extracted from the patients’ medical record and confirmed with the patient during their first study visit. The following data were collected: age, sex, HBI, recent endoscopic findings, current medication, previous IBD surgery, last use of antibiotics and/or probiotics, corticosteroids, and laxative use. 

Dietary intake was collected using prospective 3-day weighed food records. Eligible patients met with the study coordinator to receive training on how to record an accurate 3-day weighed food record. Patients were instructed to document all food and drinks consumed and preparation method on two representative weekdays, and one weekend day. The food records were then reviewed by the study dietitian (RD) with the patient to identify any missing food or drink items. 

Three-day dietary intake data were subsequently entered and analyzed using ESHA Food Analysis Software (Version 11.3 X, ESHA Food Processor Nutrition Analysis Software, Salem OR, USA) [25]. Intake data was reviewed for acceptable ranges of macro- and micronutrients. Adherence to a Mediterranean diet was measured using an adapted 13-item PREDIMED Mediterranean Diet Score (P-MDS) [26]; points were not given for red wine consumption considering the impact alcohol consumption may have on symptoms related to IBD. Each item in the P-MDS received a score of 0 or 1, and items were evenly weighted. Total P-MDS scores ranged from 0–13. 

Macro- and micronutrient intakes of CD patients were compared to population usual nutrient intakes from the Canadian Community Health Survey (CCHS; Cycle 2.2) [27]. A complete description of the sampling frame is provided in the following documentation by Statistics Canada (www.statcan.ca/cgi-bin/imdb/p2SV.pl?FunctioN = getSurvey&SDDS=5049&lang=en&db=IMDB&dbg=f&adm=8&dis=2#b3). The sampling strategy was designed to provide a representative sample based on age, sex, geography, and socioeconomic status [28]. The CCHS used interviews to collect 24-h recall data with a subsample of respondents completing a second recall. To transfer daily intake into usual intake, a measurement error model was used to reduce the effect of within-individual variance and measure between-individual variance [27]. Data were compared for the following parameters: (a) total energy intake in kilocalories (kcal); (b) protein, carbohydrate, fiber, total fat, and PUFA including omega-6 and 3, MUFA and saturated fat (SFA) as proportions of total energy intake; and (c) select micronutrients (vitamins and minerals) reported as a percentage of daily recommended intakes (DRI) and mean daily/usual intakes.

### 2.5. Statistical Analysis

Statistical analysis was carried out using SPSS (IBM Corp. Version 24.0, Armonk, NY, USA). Data were expressed as means with standard deviation (SD) or standard errors (SE) where it facilitated a comparison with normative population data. Median and interquartile ranges (IQR) were presented for P-MDS due to the variability of the data. Comparisons were done using independent sample *t*-tests to identify mean differences between males and females and between CD patients’ and population usual intakes. Chi-squared tests were conducted to identify gender differences in P-MDS for categorical variables and independent sample t-tests were used for mean P-MDS scores. Statistical significance was established at *p* < 0.05. 

## 3. Results

### 3.1. Demographic and Health

Eighty-seven patients were originally recruited to participate, but complete dietary data were only completed by sixty-seven patients. These 67 patients had a mean age of 45, and a mean BMI characterized as overweight (Table 1). Most patients (female = 88%, male = 73%) were receiving anti-TNF maintenance therapy. Patients who were not receiving anti-TNF therapies were not on other IBD specific therapies including dietary therapies. Less than half of the study participants received immunomodulators. Close to one quarter of patients had one previous small bowel resection, none had colonic resections, and no patients had an ileostomy.

### 3.2. Macronutrient Intake

Compared with the representative sample, male and female patients with CD had similar energy, protein, carbohydrate, and total fat intake (Table 2). However, fat intake from PUFA, omega-6, omega-3 in females only, and MUFA were significantly lower in patients with CD when compared to the representative sample of Albertans (Table 2). Dietary fiber intake was significantly higher in patients with CD when compared to the representative sample. 

### 3.3. Vitamin Intake

Vitamin comparisons are all listed in Table 3. Compared to the representative sample, vitamins C, D, thiamin, and niacin were significantly lower in male and female patients with CD, whereas a lower intake of riboflavin and pyridoxine was observed only in female patients with CD, and lower folate intake was noted only in males with CD. Males compared to female patients with CD had a significantly lower intake of vitamin K, and higher thiamin, niacin, pyridoxine, and biotin intakes. Compared to the DRIs, both male and female patients had inadequate intakes of vitamins A, D, E, pantothenic acid, biotin, folate, and choline, although there was a high level of variation around the mean scores. Representative sample data for some vitamins (E, K, pantothenic acid, biotin, and choline) and minerals (chromium, copper, manganese, and selenium) were not available for comparison. 

### 3.4. Mineral Intake

Compared to the representative sample, intakes of magnesium, phosphorus, zinc, and potassium was lower for both male and female CD patients, whereas sodium intake was higher in female patients (Table 4). Compared to male patients with CD, female patients had lower intakes of iron, phosphorus, and sodium. Both male and female patients with CD did not meet the DRI adequacy for calcium, chromium, magnesium, zinc, or potassium.

### 3.5. P-MDS Adherence

Few patients with CD met the P-MDS criteria for olive oil, vegetable, legume intake or consumption of sofrito sauce (made from olive oil, onions, garlic, and tomatoes, and is similar to spaghetti sauce; Table 5). One quarter of male patients and one fifth of female patients met the P-MDS criteria for fruit servings, while 36% of males and 24% of females met the P-MDS criteria for nuts. Fish and/or shellfish intake had a median intake of 0 per week and 21% of males and 32% of females ate more than three or more servings a week. Over 70% of patients met the serving recommendations for limiting butter, sugar sweetened beverages, and red or processed meat, and approximately half of the patients limited baked goods and sweets to less than three times a week. Although patients reported limiting red meat according to the P-MDS criteria (<6 oz or 175 g/day), few chose poultry over red meat on a regular basis.

## 4. Discussion

The present study is a description of the dietary patterns and food intake at one time point at a single center, in adult patients in remission with CD. To our knowledge, this was the first study to report on P-MDS in an adult cohort of patients with CD, and to examine the population data and gender differences in nutrient intakes. Major study findings identified significantly different (a) micronutrient intakes between CD patients and a representative sample of individuals; (b) micronutrient intakes between male and female CD patients; and (c) dietary patterns compared to the P-MDS recommendations. 

Few published studies have identified dietary predictors of relapse. One such study in 103 CD and ulcerative colitis (UC) patients used a cross-sectional design to evaluate the association between food intake and course of disease; half with active disease [19]. Intake of legumes and potato were inversely associated with the risk of active disease, defined as fecal calprotectin >150, with close to 80% of the highest quartile consumers of legumes at a lower risk of active disease (adjusted Odds Ratio (OR) 0.21, 95% CI 0.57–0.81). Similarly, patients consuming the highest intake of meat coincided with a higher risk of active disease (unadjusted OR 3.6). In the Tasson study [19], significant effects for fruits and vegetables on disease relapse were not identified. The duration of active inflammation before data collection prevented the authors from inferring the causality of diet determinants of relapse. It is compelling to observe that in our current study, only three individuals consumed three or more servings of legumes, and 30% of male patients demonstrated meat overconsumption. 

Similar to our study, significant micronutrient deficits were observed including suboptimal intakes of vitamins A, E, C, folate, and zinc. Vitamins C and E are antioxidants, and supplementation has previously been shown to result in a reduction of oxidative stress [32]. Zinc plays a pivotal role in wound repair, tissue regeneration, and the immune system; zinc deficiency in IBD has been associated with an increased risk of hospitalizations, surgeries, and disease related complications. Normalization of zinc was associated with improvement of these adverse clinical outcomes [33]. Limited dietary diversity including the avoidance of food groups may offer one possible explanation for the deficiency in micronutrient intake. Optimizing dietary diversity through focused nutrition counseling may lead toward an improvement in micronutrient intake. 

Compared to the representative sample, dietary MUFA intake from foods like olive and canola oils, avocado, and nuts were low in patients with CD. One recently published study showed that dietary lipid and overall energy intake in a mixed IBD sample was higher than that of the healthy control subjects, however, no differences were observed in protein and carbohydrate intake [34]. Dietary lipid composition, unfortunately, was not reported in this study, rendering the proportion of MUFA and PUFA unknown. An older study in patients stratified by disease severity using the CD Activity Index threshold of 150, showed the mean energy and macronutrient intakes were close to the recommended levels in both patients with active disease and in remission, and reported MUFA intakes of 12% [35]; the MUFA intakes in our CD patients were significantly lower than this. In rheumatoid arthritis, a high intake of MUFAs, was recently demonstrated to be an independent predictor of remission [36].

Gender differences in micronutrient intakes were observed although there was a large degree of variability in the sample. Females consumed less thiamin compared to males, however, they still met 80% of the DRI. Given the adequacy of intake, the clinical relevance of this finding is likely not significant. Males only consumed half of the recommended potassium intake, and had lower intakes than females. Both males and females had low intakes of biotin compared to DRI recommendations; however, females were significantly lower than males at 25%. The consequence and etiology for these findings are not readily apparent, although likely result from the extremely low intakes of legumes, fruit and vegetables, and nuts and seeds. Although both males and females exceeded the DRI recommendation for sodium, males had a significantly greater intake, possibly a surrogate for a greater intake of processed foods. Both males and females exceeded DRIs for sodium in the representative sample, however, to a lesser extent than our sample of patients with CD. While the dietary intake of sodium is under-reported in IBD, there are signs that show a diet enriched in sodium chloride enhances inflammatory cytokine production and exacerbates colitis in animal models [37].

The dietary composition in our patient sample differed from the P-MDS criteria. Olive oil was seldom used as the primary culinary fat of choice, and few patients in our sample met the P-MDS criteria for intake of vegetables or legumes and fruit including fruit juice, and median intakes were 50% below the recommended three or more fruit servings a day. For fruit and vegetable intake, the P-MDS criteria are similar to the North American dietary recommendations. Thirty percent of males exceeded the recommendations for red or processed meats per P-MDS criteria. Over 80% of patients reported inadequate intake of fish and nuts. 

Data evaluating the relationship between Mediterranean diets (MD) and disease characteristics (severity and relapse) in IBD are limited. Drawing from the cardiovascular literature, the incidence of major cardiovascular events was lower among participants assigned to an energy-unrestricted MD, and MD supplemented with extra-virgin olive oil or nuts when compared to those assigned to a reduced-fat diet [23]. A subsequent study identified significant reductions in plasma concentrations of high-sensitivity C-reactive protein, interleukin-6, and tumor necrosis factor in patients following a MD supplemented with extra-virgin olive oil or nuts compared to a low fat diet, identifying anti-inflammatory signals to explain the observed clinical findings [38]. Recently, Arpon et al. identified that specific components of the MD, particularly nuts and extra virgin olive oil, were able to induce methylation changes in several peripheral white blood cells, showing a role for specific fatty acids on epigenetic modulation [39]. To our knowledge, studies assessing the gut microbiome composition in long term MD consumers compared to habitual diet in humans have not yet been completed. However, a very recent study identified significant microbiome differences in non-human primates consuming MD when compared to a Western diet [40]. 

The results of our study provoke further curiosity into the relationships between dietary patterns, nutrition adequacy, and disease pathogenesis in IBD. Protective mechanistic roles for MD patterns in IBD are plausible, mediated through anti-oxidant effects, microbiome changes, and anti-inflammatory effects. Interventional studies are required to test the efficacy of MD patterns on disease severity. Promising effects on disease activity have been observed with elimination diets such as the specific carbohydrate diet [41] and the autoimmune protocol diet [42], among other test diets in the recent literature, highlighting the growing interest in undertaking intervention studies in this population of patients.

The P-MDS was useful in identifying low intakes of foods rich in the deficient micronutrients like olive oil, legumes, nuts, and fruits and vegetables. In patients with suboptimal intakes of these foods, early referral to effective nutrition intervention programs and/or supplementation should be considered. At the present time, in our center, patients are not routinely referred for dietary assessment and counseling, nor do they receive nutrition risk screening. Gastroenterologists and nurse practitioners providing care to patients with IBD at our site generally refrain from offering diet-focused therapies as a solution for disease management in the absence of overt malnutrition. We recognize that the lack of dietary focus as therapy may differ from other centers. We also recognize that patients with IBD assessed at our center are interested in dietary therapies, although the source for nutrition education is not readily apparent. Patients with IBD had a higher dietary fiber intake compared to the representative sample, and 70% met the serving recommendations to limit butter, sugar sweetened beverages, and processed meat. 

There are study limitations that should be noted. This study was conducted at a single Canadian center with a small sample, and therefore, the generalizability of the study findings may be limited. While there are limitations to the use of the 3-day food record to capture dietary intake such as biased estimates of intake due to underreporting or changes in normal dietary patterns, this validated tool has been used extensively to measure food intake in a variety of populations. Deficient dietary intake may not be synonymous with deficient total body stores of micronutrients. The current study design was unable to infer a relationship between intake and body stores. In addition, the representative sample data did not report on several micronutrients, so comparisons between patients with IBD and this population were not possible for all micronutrients. This study was underpowered to detect a medium difference between males and females and produced point estimates with a large degree of variability; therefore, results should be interpreted with caution. In addition, multiple analyses were conducted, increasing the risk of type 1 error; however, due to the exploratory nature of this study and limited sample size, a statistical correction was not made and results should be interpreted with this in mind. Additional studies with larger numbers are recommended to confirm this study’s conclusions. 

Studies such as this one are necessary to identify the need for dietary intervention and offers potential dietary targets for intervention in patients with CD in an ambulatory care setting. Future directions include a further description of dietary patterns in patients with CD and dietary intervention studies to test the feasibility, acceptability, compliance, and efficacy of a MD pattern on disease related outcomes. Dietary intervention studies may be regarded as complex interventions, and should be developed based on established frameworks that may include a needs assessment of the population, rationale for intervention development, and pilot studies to inform the details of a subsequent robust clinical trial [43].

## 5. Conclusions

Differences in dietary quality and micronutrient intakes were observed between a representative population and male and female patients with CD. Compared with a MD pattern shown to have anti-inflammatory properties, patients with CD reported food choices that promote a more restricted nutrient intake. These results suggest patients with CD should be screened for nutrient deficiencies and inadequacy of dietary patterns. Using a simple tool such as the P-MDS may be a practical and effective way to identify pro-inflammatory dietary patterns at an individual level. These data may be used to inform future dietary interventions in this population to optimize dietary intake. Further investigation into the efficacy of nutrition interventions to improve MD quality and the effect this has on clinical outcomes and nutrient deficiency is warranted. 

## Figures and Tables

**Table 1:** Patient demographics and health information.

	Female *N* = 34	Male *N* = 33
**Age in years (mean, SD)**	44.7 (14.4)	49.7 (12.7)
**BMI (kg/m^2^; mean, SD)**	27.8 (6.1)	26.7 (3.9)
**Anti-TNF *n*, (%)**	30 (88.2%)	24 (72.7%)
**IMM *n*, (%)**	15 (44.1%)	14 (42.4%)
**Previous bowel surgery *n*, (%)**	7 (20.6%)	11 (33.3%)

Abbreviations: SD: Standard Deviation, BMI: body mass index, Anti-TNF: Anti-tumor necrosis factor, IMM: Receiving immunomodulators.

**Table 2:** Macronutrient composition compared to current guidelines.

Macronutrient	DRI Acceptable Macronutrient Distribution Range (AMDR) and Adequate Intake (AI) [29]	Academy of Nutrition and Dietetics [30]	Crohn’s Patients (*N* = 67; Mean ± SE)		Representative Sample (*N* = 1547; Mean ± SE) [27]	
			M (*N* = 33)	F (*N* = 34)	M (*N* = 721)	F (*N* = 826)
Total energy intake (kcal/d)	Male = 662 − (9.53 × age (y)) + PA × {(15.91 × weight (kg)) + (539.6 × height (m))}, Female/Women = 354 − (6.91 × age (y)) + PA × {(9.36 × weight (kg)) + (726 × height (m))}		2358 (95.3)	1881 (86.5)	2346 (61)	1730 (42)
Protein (% total energy)	10–35% total energy		18.3 (1.0)	18.0 (0.7)	17.0 (0.4)	16.8 (0.3)
Carbohydrate (% total energy)	45–65% total energy		47.1 (1.5)	48.1 (1.1)	48.7 (0.9)	48.5 (0.6)
Fiber (g/day) ^	M: 30–38 F: 21–25		22.8 (1.4) *	20.9 (1.6) *	19.2 (0.6)	13.9 (0.5)
Total fat (% total energy)	20–35% total energy		33.7 (1.2)	34.3 (1.0)	31.0 (0.8)	32.4 (0.6)
PUFA (% total energy)	5–10% total energy	3–10% total energy	4.5 (0.4) *	3.9 (0.3) *	5.6 (0.2)	5.6 (0.1)
Omega-6 (linoleic)	5–10% total energy	3–10% total energy	3.3 (0.4) *	2.8 (0.2) *	4.5 (0.1)	4.5 (0.2)
Omega-3 (α-linolenic)	0.6–1.2% total energy	0.6–1.2% total energy	0.6 (0.15)	0.5 (0.09) *	0.8 (0.04)	0.8 (0.02)
MUFA (% total energy)	No AI level	15–20% total energy	8.2 (0.6)*	7.1 (0.5) *	12.6 (0.4)	12.8 (0.2)
SFA (% total energy)	As low as possible	7–10% of total energy <7% to reduce CVD risk 5–6% to lower lipids	10.7 (0.4)	11.1 (0.5)	9.8 (0.2)	10.7 (0.3)
TransFA (% total energy)	As low as possible	<1% total energy	0.4 (0.1)	0.3 (0.1)	unavailable	

Abbreviations: Dietary reference intakes (DRI), Male (M), Female (F), Polyunsaturated fatty acids (PUFA), Monounsaturated fatty acids (MUFA), Saturated Fatty Acids (SFA), Cardiovascular disease (CVD), Males (M), Females (F). TransFA, Trans Fatty Acids. * Indicates significant differences between patients with Crohn’s compared to Albertan population, results are gender specific (*p* < 0.05). ^ Higher values are for adults 19–50 years.

**Table 3:** Vitamin intake compared to a representative population.

Vitamins	DRI Adequate Intake/day (AI) [31]		Crohn’s Patients (*N* = 67)				Representative Sample (*N* = 1547) [27]	
	Males	Females	M (*N* = 33)% of DRI (M ± SD)	F (*N* = 34)% of DRI (M ± SD)	M (*N* = 33) Daily intake (M ± SE)	F (*N* = 34) Daily intake (M ± SE)	M (*N* = 721) Usual intake (M ± SE)	F (*N* = 826)Usual intake (M ± SE)
Vitamin A RAE μg	900	700	69 (55)%	97 (162)%	609 (86)	682 (195)	667 (33)	577 (28)
Vitamin D μg ^@^	15–20		21 (20)%	16 (17)%	3 (0.5) **	2.5 (0.4) **	5.9 (0.3)	5.0 (0.3)
Vitamin E α-tocopherol mg	15		48 (59)%	32 (28)%	5 (1.5)	7.1 (0.7)	n/a	n/a
Vitamin K μg	120	90	52 (46)% *	106 (101)% *	61 (9.7)	97 (15.7)	n/a	n/a
Vitamin C mg (*N* = 1484) Vitamin C smokers mg (*N* = 679)	90 125	75 110	121 (78)%	108 (84)%	106 (12) **	82 (11) **	143 (8) 92 (7)	113 (4) 102 (8)
Thiamin, B1 mg	1.2	1.1	115 (60)% *	82 (41)% *	1.4 (0.12) **	0.9 (0.08) **	2.0 (0.07)	1.4 (0.04)
Riboflavin, B2 mg	1.3	1.1	141 (67)%	113 (52)%	1.8 (0.15)	1.3 (0.10) **	2.1 (0.07)	1.6 (0.05)
Niacin, B3 NE	16	14	212 (113)% *	161 (76)% *	34 (2) **	23 (2) **	46 (2)	32 (1)
Pantothenic Acid, B5 mg	5		87 (46)%	68 (35)%	4.4 (0.4)	3.4 (0.3)	n/a	n/a
Pyridoxine, B6 mg ^t^	1.3–1.7	1.3–1.5	119 (64)% *	86 (45)% *	1.8 (1.0)	1.2 (0.7) **	2.1 (0.1)	1.6 (0.1)
Biotin, B7 mg	30		47 (42)% *	25 (19)% *	14 (2.2)	8 (1.0)	n/a	n/a
Folate, B9 DFE μg	400		72 (32)%	61 (59)%	287 (34) **	244 (33)	488 (15)	325 (41)
Cobalamin, B12 μg	2.4		177 (123)%	130 (109)%	4.2 (0.5)	3.1 (0.5)	4.9 (0.3)	3.5 (0.2)
Choline mg ^	550	425	43 (26)%	39 (23)%	229 (25)	165 (17)	n/a	n/a

Abbreviations: Retinol Activity Equivalents (RAE), Dietary Folate Equivalents (DFE), Not available (n/a). ^@^ Higher values for greater than 70 years. Does not include values for children or pregnant females. ^t^ Higher values for 51 years and older. ^ Choline requirements may be met by endogenous production and food sources may not be required. * Indicates a significant difference in %DRI between males and females (*p* < 0.05). ** Indicates a significant difference between patients with Crohn’s and representative sample intakes, specific to gender (*p* < 0.05).

**Table 4:** Mineral intake compared to a representative population.

Minerals	DRI Adequate Intake/Day (AI) [31]		Crohn’s Patients, (N = 67)				Representative Sample (N = 1547) [27]	
	Males	Females	M (N = 33) % of DRI (M ± SD)	F (N = 34) % of DRI (M ± SD)	M (N = 33) Daily intake (M ± SE)	F (N = 34) Daily intake (M ± SE)	M (N = 721) Usual intake^2^ (M ± SE)	F (N = 826) Usual intake^2^ (M ± SE)
Calcium mg/d ^@^	1000–1200		87 (49)%	73 (42)%	906 (97)	785 (76)	890 (38)	799 (35)
Chromium μg/d ^t^	35–30	25–20	18 (50)%	8 (6)%	5.8 (3.1)	1.7 (0.3)	n/a	n/a
Copper μg/d	900		110 (63)%	93 (69)%	989 (99)	837 (106)	n/a	n/a
Iron mg/d^	8	8–18	187 (102)% *	118 (86)% *	17 (1.6)	13 (1.1)	16 (0.5)	11 (0.3)
Magnesium mg/d ^#^	400–420	310–320	63 (33)%	60 (34)%	256 (23) **	191 (19) **	352 (9)	274 (7)
Manganese mg/d	2.3	1.8	197 (306)%	114 (64)%	4.5 (1.2)	2.1 (0.2)	n/a	n/a
Phosphorus mg/d	700		144 (68)% *	111 (54)% *	1030 (79) **	777 (65) **	1500 (44)	1152 (33)
Selenium μg/d	55		151 (81)%	134 (78)%	83 (7.8)	74 (7.4)	n/a	n/a
Zinc mg/d	11	8	83 (38)%	72% (34)	9 (0.7) **	6 (0.5) **	13 (0.5)	10 (0.3)
Potassium mg/d	4700		54 (30)%	44 (31)%	2532 (246) **	1880 (157) **	3355 (103)	2657 (62)
Sodium mg/d	1200–1500 ^+^		169 (74)% *	135 (51)% *	3880 (296)	3101 (203) **	3543 (120)	2550 (75)

Abbreviations: Not available (n/a); * Indicates a significant difference between males and females (*p* < 0.05). ** Indicates a significant difference between Crohn’s patients and the Albertan population, gender specific (*p* < 0.05). ^@^ Higher values for greater than 70 years. Does not include values for children or pregnant females. ^t^ Higher values for 51 years and older. ^ Higher values are for menstruating females. ^#^ Higher values for 31 years and older. ^+^ Lower values for 51 years and older.

**Table 5:** Predimed Mediterranean Diet (P-MDS) adherence scores.

P-MDS Adherence Criteria [26]	Canada Food Guide 1-Serving Size and P-MDS Answers	Canada Food Guide Servings (Median ± IQR)		Percent Meeting P-MDS Criteria *n* (%)	
		Male	Female	Male	Female
Olive oil as main culinary fat	Yes, No answer	n/a	n/a	2 (6)	0 (0)
≥4 tbsp olive oil per day	1 Tbsp (15 mL)	n/a	n/a	0 (0)	0 (0)
≥5 servings vegetables with ≥2 servings as raw leafy vegetables per day	½ cup (125 mL) fresh, frozen, canned vegetables 1 medium vegetable 1 cup (250 mL) raw or leafy vegetables	total 1.0 (0.4–1.7) leafy 0.0 (0.0–0.5)	total 1.9 (1.0–3.0) leafy 0.5 (0–1.0)	0 (0)	1 (3)
≥3 servings fruit and unsweetened fruit juice per day	½ cup (125 mL) fresh, frozen, canned fruit 1 medium fruit ½ cup (125 mL) unsweetened fruit juice	1.8 (1.3–3.3)	1.4 (0.9–2.6)	9 (27)	7 (21)
<2 servings of red meat, hamburger, or processed meat such as ham, sausage, etc. per day	85 g (3 oz) meat ½ cup (125 mL)	1.7 (1.0–2.0)	0.7 (0.3–1.5)	23 (70)	28 (82)
<1 serving of butter, hydrogenated margarine or cream per day	1 Tbsp (15 mL)	n/a	n/a	27 (82)	27 (79)
<1 sugar sweetened beverage per day	Yes, No answer	n/a	n/a	31 (94)	27 (79)
≥3 servings beans, peas, lentils per week	¾ cup (175 mL)	0.0 (0.0–0.0)	0.0 (0.0–0.2)	3 (9)	3 (9)
≥3 servings fish and/or shellfish per week	CFG = 85 g (3 oz.) 100 g (3.5 oz) fish 200 g (7.0 oz) shellfish	fish 0.0 (0.0–0.0) seafood 0.0 (0.0–0.0)	fish 0.0 (0.0–3.1) seafood 0.0 (0.0–0.0)	7 (21)	11 (32)
Eat <3 times a week baked goods, sweets, pastries or candy	Yes, No answer	n/a	n/a	19 (58)	19 (56)
≥3 servings nuts per week	¼ cup nuts or whole large seeds (30 g) 2 Tbsp (30 mL) nut and seed butters or small/ground seeds	0.0 (0.0–5.4)	0.4 (0.0–2.6)	12 (36)	8 (24)
Choose chicken, turkey or rabbit more often than veal, pork, hamburger or sausage	Yes, No answer	n/a	n/a	12 (36)	18 (53)
Consume sofrito sauce ≥2 times per week	Yes, No answer	n/a	n/a	4 (12)	11 (32)
**Total mean MDS Score (possible score out of 13)**				4.5 (1.1)	4.7 (1.8)

Abbreviations: interquartile range (IQR); No significant differences in P-MDS criteria were evident using chi-square tests between males and females for categorical variables and independent sample t-tests for mean MDS score (*p* < 0.05).
